# EEG slow-wave mediates the fragmentation and coupling of cortical networks in propofol-induced general anesthesia

**DOI:** 10.1186/1471-2202-16-S1-P231

**Published:** 2015-12-18

**Authors:** Kaier Wang, Moira L Steyn-Ross, Alistair Steyn-Ross, Marcus T Wilson, Jamie W Sleigh

**Affiliations:** 1 School of Engineering, The University of Waikato, Hamilton, 3240, New Zealand; 2 Waikato Clinical School, The University of Auckland, Waikato Hospital, Hamilton, 3240, New Zealand

## 

Electroencephalogram (EEG) recorded from propofol-induced general anesthesia is characterized by large amplitude slow-waves (0.1--1.5 Hz). Clinically, these lowest frequency components of the EEG signal become dominant over other higher frequency components during and after loss of consciousness [1]. However, it remains unclear how these slow oscillations are produced and to what extent they reflect changes in cortical network connectivity. Modeling anesthesia as a moderate reduction in interneuronal gap-junction coupling, a recent theoretical work by Steyn-Ross et al [2] predicts emergence of anesthetic slow-waves with chaotic dynamics. In the modeled anesthesia state, the weakened gap-junction coupling supports a codimension-2 bifurcation point where competing Turing (space) and Hopf (time) dynamics coexist, signifying spontaneous symmetry-breaking instabilities in the firing behavior of cortical neurons. Further, these chaotic slow-waves are found to perturb the neuronal coupling across the cortex, leading to a dramatic drop in global phase-coherence compared to its high level during consciousness. In this study, we analyze clinically-recorded EEG data to examine the model prediction for changes in phase-coherence between pairs of EEG channels in the sub-delta band during propofol anesthetic induction. Our study finds a coherence decrease in the frontal and occipital regions (see left panel of Figure 1), as well in the connection between them. Concomitantly, more strongly coupled neuronal activities are disclosed in the temporal-frontal, temporal-occipital and left-right temporal regions (right panel). Our clinical observation of reduced EEG coherence is consonant with cortical model predictions. However, our EEG study indicates that the coherence alternation is regional in nature, while the cortical model describes a spatially-uniform trend. Moreover, we did not find any theoretical prediction for the left- and right-temporal increased-coherence patterns. As the cortical model by Steyn-Ross et al. is spatially homogenous, i.e., there are no explicit front-to-back or right-to-left directionality, it is unable to produce regional coherence changes. It appears that the Steyn-Ross cortical model best represents the cortical dynamics in the frontal region.

**Figure 1:**
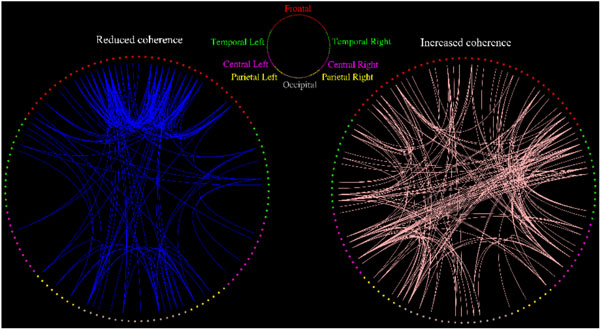
**Correlation representations showing electrode pairs with significantly reduced (left, blue) or increased (right, pink) phase-coherence for sub-delta band (0.05-1.5 Hz) EEG induced by propofol anesthesia**. Only electrode pairs (from 128 scalp electrodes) showing significant (*p *< 0.025) change in phase coherence are connected with lines.

## References

[B1] Lewis LD Weiner VS Mukamel EA Donoghue JA Eskandar EN Madsen JR Rapid fragmentation of neuronal networks at the onset of propofol-induced unconsciousness Proc. Natl. Acad. Sci. U.S.A 2012 109 E3377 E3386 23129622 10.1073/pnas.1210907109 PMC3523833

[B2] Steyn-Ross ML Steyn-Ross DA Sleigh JW Interacting Turing-Hopf instabilities drive symmetry-breaking transitions in a mean-field model of the cortex: a mechanism for the slow oscillation Phys. Rev. X 2013 3 021005

